# Differential expression of GluN2 NMDA receptor subunits in the dorsal horn of male and female rats

**DOI:** 10.1080/19336950.2020.1871205

**Published:** 2021-01-28

**Authors:** Santa Temi, Christopher Rudyk, Jennifer Armstrong, Jeffrey A. Landrigan, Chris Dedek, Natalina Salmaso, Michael E. Hildebrand

**Affiliations:** a Department of Neuroscience, Carleton University, Ottawa, Canada; b Neuroscience Program, Ottawa Hospital Research Institute, Ottawa, ON, Canada

**Keywords:** GluN2, GluN2A, GluN2B, GluN2D, NMDA receptor, CGRP, dorsal horn, sex, spinal cord, laminae

## Abstract

N-methyl-D-aspartate receptors (NMDARs) are excitatory ionotropic glutamate receptors expressed throughout the CNS, including in the spinal dorsal horn. The GluN2 subtypes of NMDAR subunit, which include GluN2A, GluN2B, and GluN2D in the dorsal horn, confer NMDARs with structural and functional variability, enabling heterogeneity in synaptic transmission and plasticity. Despite essential roles for NMDARs in physiological and pathological pain processing, the distribution and function of these specific GluN2 isoforms across dorsal horn laminae remain poorly understood. Surprisingly, there is a complete lack of knowledge of GluN2 expression in female rodents. We, therefore, investigated the relative expression of specific GluN2 variants in the dorsal horn of lumbar (L4/L5) spinal cord from both male and female rats. In order to detect synaptic GluN2 isoforms, we used pepsin antigen-retrieval to unmask these highly cross-linked protein complexes. We found that GluN2B and GluN2D are preferentially localized to the pain-processing superficial regions of the dorsal horn in males, while only GluN2B is predominantly localized to the superficial dorsal horn of female rats. The GluN2A subunit is diffusely localized to neuropil throughout the dorsal horn of both males and females, while GluN2B and GluN2D immunolabelling are found both in the neuropil and on the soma of dorsal horn neurons. Finally, we identified an unexpected enhanced expression of GluN2B in the medial division of the superficial dorsal horn, but in males only. These sex-specific localization patterns of GluN2-NMDAR subunits across dorsal horn laminae have significant implications for the understanding of divergent spinal mechanisms of pain processing.

## Introduction

Poorly managed pain is a global health concern. Women, in particular, show a high prevalence and sensitivity to both visceral and somatic pain and an increased tendency to develop certain chronic pain diseases [1,2]. To develop better treatment approaches for these conditions, the molecular underpinnings of pain processing need to be fully characterized. Unfortunately, the vast majority of investigations into the molecular and cellular mechanisms of spinal nociceptive processing have been conducted in male rodent models of pain only. Direct comparison of the molecular determinants of spinal excitability between males and females is therefore essential to understand which potential pain targets are conserved or diverge across sex.

The dorsal horn of the spinal cord is a critical site for pain transmission and modulation in the central nervous system (CNS). Based on the Rexed classification of transverse spinal sections [3], two main divisions of the dorsal horn include the superficial dorsal horn (SDH), composed of laminae I and II, and the deep dorsal horn (DDH), composed of laminae III to VI. The latter is marginally involved in processing noxious stimuli, with neural circuits that are primarily dedicated to processing non-nociceptive somatosensory inputs [4]. In contrast, the SDH consists of a complex nociceptive network that integrates sensory signals from peripheral nociceptive afferents with local interneuron processing as well as descending efferent modulation from the brain [5]. Among the afferents that innervate the SDH, peptidergic nociceptive fibers that release calcitonin gene-related peptide (CGRP) terminate onto lamina I and the outer portion of lamina II [6], with some collaterals that reach deeper laminae [7]. Therefore, CGRP is an excellent marker of the outermost, nociceptive portion of the SDH.

Glutamate is the major excitatory neurotransmitter of spinal nociception, with activation of glutamatergic N-methyl-D-aspartate receptors (NMDARs) being a central mediator of dorsal horn plasticity in both physiological and pathological pain states [8–10]. NMDARs are tetra-heteromeric complexes consisting of a variety of possible combinations of GluN1, GluN2, and/or GluN3 subunits [11]. There are four genetically encoded isoforms of the GluN2 subunit, GluN2A, GluN2B, GluN2C, and GluN2D, and the differential composition of specific GluN2 subtypes in NMDAR complexes enables diversity in the function and regulation of NMDARs across CNS regions and neuron subpopulations. For example, GluN2A- and GluN2B-containing NMDARs are differentially phosphorylated by Src family kinases and mediate distinct forms of plasticity in brain regions such as the hippocampus [12]. However, despite our understanding of the specific roles of individual GluN2-NMDAR subunits in mediating synaptic transmission and plasticity in the brain, this knowledge is almost exclusively based on studies conducted in male or unsexed animals. Given that divergent molecular mechanisms of spinal pain processing are being identified between sexes [13], it is critical to systematically characterize the expression profiles of NMDAR subunits in the spinal cord (and brain) in both males and females.

Based on available studies in male rodents, three of the four GluN2 isoforms are expressed in the dorsal horn – GluN2A, GluN2B, and GluN2D [9]. Both transcript [14] and protein [15] levels for GluN2C have been found to be either negligible or undetected in the dorsal horn, including recent findings using single-cell sequencing approaches [16]. Despite molecular and functional evidence for GluN2A, GluN2B, and GluN2D expression in the dorsal horn [17–24], there are considerable discrepancies on the relative localization of these subunits across dorsal horn laminae as well as their contributions to responses at dorsal horn synapses. Reconciling results between these individual studies is often challenging given different types of functional measures (e.g. spontaneous and evoked synaptic responses versus agonist-evoked responses), a lack of investigation into contributions from all possible GluN2 subunit types, and variations between the subpopulations of dorsal horn neurons under study. An unbiased approach is therefore urgently needed to compare the relative expression and localization of GluN2A, GluN2B, and GluN2D across the dorsal horn in both sexes. Given the emerging roles for specific NMDARs, such as GluN2B, in pathological spinal pain processing [25,26], this information will provide a foundational understanding on baseline expression of NMDAR subunits for future studies into how their dysregulation may drive chronic pain.

To systematically characterize the expression of the three relevant GluN2-NMDAR subunits within the SDH versus DDH regions of the dorsal horn, we performed qualitative and quantitative immunohistochemistry on fixed spinal sections of male and female juvenile rats. Through a combination of pepsin antigen-retrieval and signal amplification techniques, we were able to detect GluN2 subunits at synaptic, extrasynaptic, and somatic loci within the dorsal horn. Finally, we examined the relative distribution of these GluN2A, GluN2B, and GluN2D NMDAR subunits across the mediolateral axis of the SDH.

## Materials and methods

### Animals

Male and female postnatal day 21 (p21) Sprague-Dawley rats were purchased from Charles Rivers Laboratories. The experimental procedures were approved by the Carleton University Animal Care Committee and conducted in accordance with the guidelines of the Canadian Council for Animal Care.

### Spinal cord isolation and preparation

Twelve Sprague-Dawley juvenile (p21) rats of both sexes (n = 6 male, n = 6 female) were anesthetized with an intraperitoneal (ip) injection of 3 g/kg urethane (Sigma-Aldrich). Lumbar spinal cord segments were dissected through ventral laminectomy according to a procedure previously described [20]. The collected spinal cords were immediately placed in an ice-cold, oxygenated, sucrose protective solution containing 50 mM sucrose, 92 mM NaCl, 17 mM D-glucose, 26 mM NaHCO_3_, 5 mM KCl, 1.25 mM NaH_2_PO_4_, 0.5 mM CaCl_2_, and 7 mM MgSO_4_. All the meninges and blood vessels were quickly removed from the meninges and blood vessels spinal cord segments. The tissues were fixed in 4% paraformaldehyde (PFA) for 24 h at 4°C, then placed in a first step of 10% sucrose for 24 h, a second step of 10% sucrose for at least 6 h, and in 30% sucrose solutions (in PB) for 72 h at 4°C. Spinal cord tissues were embedded in OCT using cryo-molders and then flash frozen through submersion in isopentane that was supercooled by liquid nitrogen. The embedded spinal cords were then stored at −80°C up until sectioning with a microtome cryostat (ThermoScientific). The spinal lumbar segments were sliced into 25 µm thick transverse sections. Each section was serially mounted onto pre-treated slides (FisherBrand^TM^ SuperFrost^TM^ Plus) to obtain approximately the same set of L1 to L6 spinal cord sections on each slide.

### Immunohistochemistry

To study the relative expression of GluN2A, GluN2B, and GluN2D isoforms in the SDH versus the DDH regions, we conducted immunohistochemistry experiments with double labeling to detect both the SDH and the receptor subunit of interest. Calcitonin gene-related peptide (CGRP) was used as the marker of the pain-processing SDH (laminae I/II) since it labels the nociceptive afferent peptidergic fibers that selectively innervate this region [27–29].

We applied a modified immunohistochemistry technique based on pepsin treatment to unmask and retrieve the antigen. The pre-treatment of the sections with pepsin allows the detection of the GluN2 subunits, including the subunits localized at synapses. Pepsin exposes the epitope of cross-linked proteins, such as those at postsynaptic densities, and facilitates the binding of the antibody to the target [17]. Therefore, after incubation in a peroxidase solution (50% methanol, 1.8% hydrogen peroxide in PBS) for 30 minutes at room temperature (RT), we treated the slides with pre-warmed pepsin at 37°C for 5 minutes. Immediately after, the sections were washed 3 times (x 5 min) with PBS. The sections were incubated with a blocking solution containing 5% NGS, 0.3% Triton X-100 and 0.3% BSA (in PBS) for 1 h. This step was followed by incubation with a mixture of primary antibodies (see below and Table 1 for antibody characteristics and dilution factors) against either GluN2A and CGRP, GluN2B and CGRP, or GluN2D and CGRP at RT, overnight. The primary antibodies were diluted in the same blocking solution used previously.

**Table 1.** List of antibodies used for immunohistochemistry

Antibody	Dilution	Manufacturer	Catalog #
Rabbit anti-GluN2A	1:2000	Alomone Labs	ACG-002
Rabbit anti-GluN2B	1:1000	Alomone Labs	ACG-003
Rabbit anti-GluN2D	1:1000	Alomone Labs	ACG-020
Mouse anti-CGRP	1:5000	Sigma-Aldrich	C-7113
Goat anti-mouse IgG (H + L) AlexaFluor647	1:1000	ThermoFisher Scientific	A-21235
Goat anti-rabbit IgG (H + L) HRP	1:500	Jackson ImmunoResearch Laboratories	111–035-003

The next day, spinal cord sections were rinsed in PBS (3 times x 5 min) and incubated in secondary antibodies, diluted in the blocking solution, for 2 h. The mixture of secondary antibodies used was the following: goat anti-mouse AlexaFluor647 (1:1000; Invitrogen, A21235) and goat anti-rabbit IgG conjugated with horseradish peroxidase (HRP) H + L (1:500; Jackson ImmunoResearch Laboratories). After washes in PBS, a tyramide signal amplification (TSA) reaction was performed using the signal amplification fluorescent kit conjugated with the fluorophore cyanine 3 (cy3) (TSA plus cyanine 3 system, Perkin Elmer, Inc., NEL744001KT). A dilution factor of 1:50 in the TSA amplification buffer was used. The amplification time was 2.5 min (GluN2A) or 7.5 min (GluN2B and GluN2D). The resultant enzymatic reaction allows for cy3 to be deposited and thus, the optimal detection and visualization of labeled NMDAR subunits [30,31]. A Hoechst 33258 Staining Dye Solution (1:1000; Abcam, ab228550) was also used to stain for nuclei (data not shown). Slides were then cover-slipped in Fluoromount^TM^ Aqueous Mounting Medium (Sigma, F4680-25ML) prior to imaging.

All immunohistochemistry experiments and measures were conducted in triplicate or quadruplicate and the raw OD values were averaged for each animal.

### Antibody details

To selectively label the GluN2A NMDAR subunit we used a polyclonal antibody (Alomone Labs, #ACG-002) raised in rabbit. This antibody recognizes the extracellular, N-terminus, sequence GHSHDVTERELRN(C) corresponding to the amino-acid residues 41–53 of the rat GluN2A. The peptide is homologous to the mice and humans’ peptide and, therefore, can be used to detect GluN2A in all of these species. This specific antibody has been used to successfully label GluN2A in previous published immunohistochemistry [32], cytochemistry [33–35], and western blot experiments.

To detect the GluN2B subunit, we used a polyclonal antibody (Alomone Labs, #AGC-003) raised in rabbit. It recognizes the extracellular, N-terminus, sequence (C)NTHEKRIYQSNMLNR corresponding to the amino-acid residues 323–337 of rat GluN2B. This GluN2B antibody has been successfully used in immunohistochemistry and cytochemistry experiments [32,35,36] as well as in western blot studies.

To label the GluN2D subunit we used a polyclonal antibody raised in rabbit (Alomone Labs, #AGC-020). It recognizes the N-terminus sequence CRTQNRTHRGESLHR corresponding to the amino-acid residues 345–359 of the rat GluN2D. This GluN2D antibody has been successfully employed in both immunohistochemistry and cytochemistry experiments [37,38].

In order to detect CGRP, we used a monoclonal antibody raised in mouse (Sigma-Aldrich, C7113), which recognizes 10 amino-acids within the C-terminus of α-CGRP of the rat.

### Image acquisition

All immunohistochemistry sections were imaged on a confocal Zeiss LSM 800 Airyscan microscope at 20x objective magnification with Zeiss ZEN 3.1 imaging software. Stacks of horizontal plane images and orthogonal projections of the entire dorsal horn were acquired for each L4/L5 lumbar section in triplicate or quadruplicate. The regions corresponding to the SDH and DDH laminae were tiled. All the settings (laser power and gain) were maintained the same for each experiment.

### Analysis and quantification of GluN2 fluorescence

In order to specifically identify and analyze the L4/L5 segments of the rat lumbar spinal cord, we visualized the spinal cord segments under a light microscope before imaging. L4 and L5 spinal sections were identified through reference to cytoarchitectural hallmarks using a rat spinal cord atlas [39]. We could distinguish L4/L5 sections largely based on gray and dorsal column white matter anatomical features, as well as the outer diameter of the spinal cord. L4 and L5 transverse section segments were slightly wider than L1, L2, L3 segments and much wider than the L6 segment. Moreover, the morphology of the gray and white matters varied along the lumbar segments as well. The dorsal horns appeared similarly rounded and curved (medially and laterally) in every lumbar segment as opposed to the ventral horns of L4, L5, and L6 which presented two main caudolateral enlarged areas with two distinguishable clusters of neurons. The dorsal column white matter of L1/L2 and L3/L6 morphologically resembled a heart and an inverted cone with rounded bases, respectively. In contrast, the dorsal column white matter of L4 and L5 appeared very narrow and extended longitudinally toward the central canal. To confirm successful segment identification, two experimenters independently classified the same lumbar sections as L4/L5.

Here we analyzed the optical density (OD) of GluN2 immunoreactivity in the CGRP-positive (CGRP+) SDH versus the remaining DDH. Despite the majority of afferent CGRP fibers terminating in the SDH, a few CGRP fibers penetrate and synapse into the deeper laminae (laminae III–VI), resulting in minor positive CGRP staining in the DDH. For simplicity, given that the strongest CGRP staining is visualized in the SDH, we referred to the SDH as the CGRP+ region, whereas we referred to the DDH as the CGRP-negative (CGRP−) region. We selected the CGRP+ area by drawing a contour along the dorsal edge of the dorsal horn and then along the more ventral border of the main CGRP-positive staining (excluding the dorsal root and the deeper laminae). We then made a second selection corresponding to the CGRP− area, which terminates in proximity of the beginning of the central canal. To assess the relative expression of the GluN2-NMDAR subunits, we measured the OD of the cy-3 stained areas in the CGRP+ and CGRP− regions using the software Fiji ImageJ. We normalized these OD values to their respective areas. Furthermore, we subsequently normalized OD/area values to the background (BG)/area values by measuring the OD within a square selection (55 x 55 μm) positioned in the deep dorsal column of white matter where there was minimal punctate GluN2-positive signal. We measured the BG values in the white matter instead of the gray matter as the GluN2 subunit staining was extensive throughout the gray matter regions and so this prevented the selection of a GluN2-negatively stained BG region in the gray matter.

In all of our experiments, we selected either the right or the left side of the dorsal horn for quantification. The selection criteria were based primarily on the integrity and quality of the fixed spinal cord tissue section. When the integrity of the section was equal for both the left and right dorsal horn, we randomly selected one of them before GluN2 staining was analyzed.

To measure the GluN2 subunit distribution across the mediolateral axis, we placed oval-shaped selections (40 x 100μm) along the most marginal edge of the CGRP+ region in the medial, central, and lateral regions of either the left or right dorsal horn. The medial and lateral ovals were positioned approximately at the “corners” where the dorsal horn curves medially and laterally, whereas the central oval was positioned at the midline of the SDH mediolateral axis.

## Statistics

IBM SPSS Statistics software was used for statistical analysis. To assess the normality of the data, Shapiro-Wilk tests were performed. As appropriate, T-tests and one-way ANOVA tests for paired or independent samples were performed to test for significant differences between groups. The results are presented as average ± standard error of the mean (SEM). P < 0.05 was considered statistically significant.

## Results

### GluN2B and GluN2D isoforms are predominantly localized in the SDH vs the DDH of male rats

**Figure 1.**
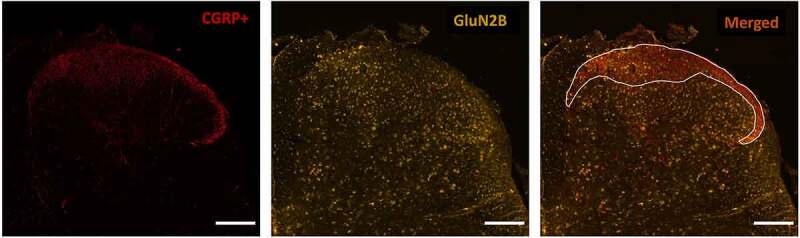
Immunohistochemical staining approach to identify the CGRP+ SDH region and study GluN2 subunit distribution. Left) Laminae I and IIo of the superficial dorsal horn (SDH) are immunopositive for punctate CGRP (red) staining, labeling the relatively nociceptive-specific marginal region of the SDH. Middle) Co-labeling with an antibody against a specific GluN2 subunit (GluN2B here, yellow) is performed on the same spinal sections stained for CGRP. Right) Overlay of CGRP and GluN2B immunostaining. The marginal SDH that is CGRP-positive is outlined (white line), and then GluN2 subunit immunoreactivity is described and quantified in this CGRP+ SDH region in comparison to the remaining deep dorsal horn (DDH) laminae. Scale bar: 200 μm

To investigate the expression of GluN2-NMDAR subunits across the SDH and DDH in L4/L5 lumbar spinal cord segments, we co-stained spinal sections with selective antibodies against specific GluN2 subtypes as well as a marker of the nociceptive SDH, CGRP (Figure 1). By performing staining against GluN2A, GluN2B, and GluN2D NMDAR subunits in parallel for both male and female juvenile (p21) rats, we were able to systematically compare patterns of NMDAR subunit expression across dorsal horn laminae and sex.

**Figure 2.**
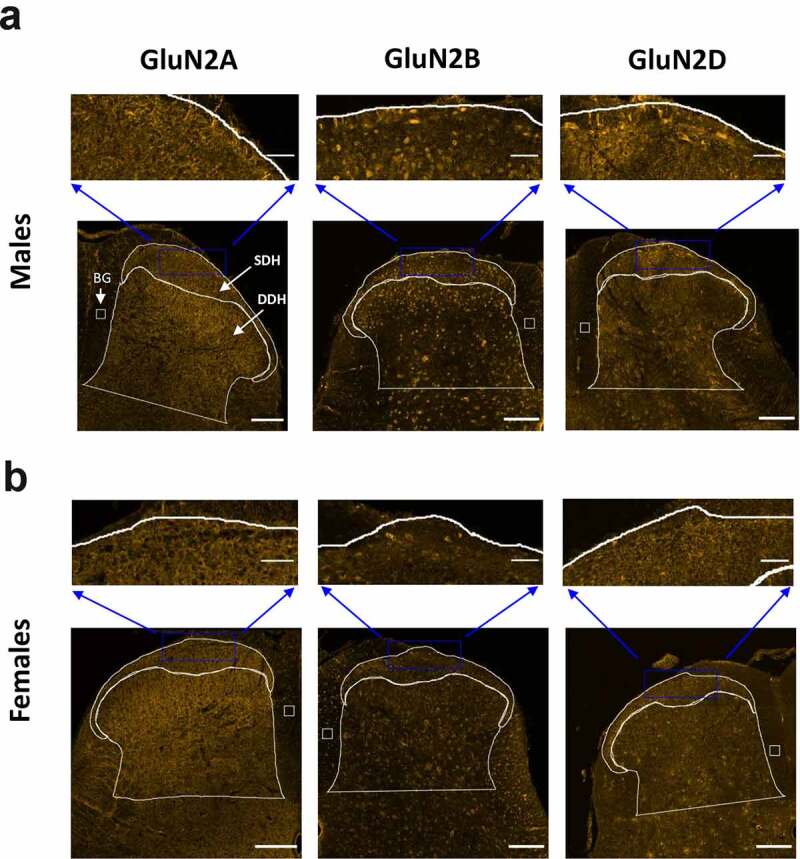
Immunolabelling of GluN2A, GluN2B and GluN2D subunits in the SDH and DDH of male and female rats. The CGRP+ marginal region of the SDH as well as the remaining DDH was outlined as described in Figure 1. For immunoreactive quantification in subsequent figures, SDH and DDH staining (per area) were normalized to background (BG) signal in a boxed region of the immuno-negative dorsal column. A, bottom) Representative confocal images (20x objective) illustrating the fluorescence staining patterns for GluN2A (left), GluN2B (middle) and GluN2D (right) in male juvenile rats. Scale bar: 200 μm. A, top) Higher magnification insets illustrating the distribution of immunoreactivity in the marginal SDH for all three GluN2 subtypes. GluN2A immunoreactivity is uniformly distributed across the neuropil of the SDH and DDH laminae, while GluN2B and GluN2D signal is also widespread but with differential expression in SDH versus DDH laminae, with staining of both neuropil and cell bodies. Scale bar: 50 µm. B, bottom) Representative confocal images (20x objective) illustrating the fluorescence staining patterns for GluN2A (left), GluN2B (middle) and GluN2D (right) in female juvenile rats. Scale bar: 200 μm. A, top) Higher magnification insets illustrating the distribution of immunoreactivity in the marginal SDH for all three GluN2 subtypes. GluN2A immunoreactivity is distributed mainly in the neuropil of the SDH and DDH laminae, while GluN2B and GluN2D signal demonstrates staining of both neuropil and cell bodies in the SDH and DDH. Scale bar: 50 µm

Starting with the staining results from male rats (Figure 2(a)), we found that the pattern of immunoreactivity across the dorsal horn varied for each individual GluN2 subunit. Qualitatively, the immunoreactivity for GluN2A was intense, localized mainly to diffuse neuropil, and evenly dispersed across the laminae of both the CGRP+ SDH and the DDH (Figure 2(a) left, and Suppl. Figure 1). The GluN2B NMDAR subunit was also found throughout the SDH and DDH; however, the signal was more concentrated in the SDH compared to the DDH (Figure 2(a) middle, and Suppl. Figure 3). In contrast to the homogenous distribution of GluN2A, the expression of GluN2B was strongly localized to the soma of cells as well as to the punctate labeling of neuropil (Figure 2(a) middle, top). GluN2B-positive cell bodies were also identified in the dorsal column white matter region, suggesting that cells outside the dorsal horn gray matter express this NMDAR isoform. The expression pattern for GluN2D was similar to that of GluN2B, but with less pronounced differential expression (Figure 2(a) right, Suppl. Figure 5). GluN2D immunoreactivity was found at both cell bodies and diffuse neuropil, with a slight increase in the intensity of overall expression in the SDH compared to the DDH.

**Figure 3.**
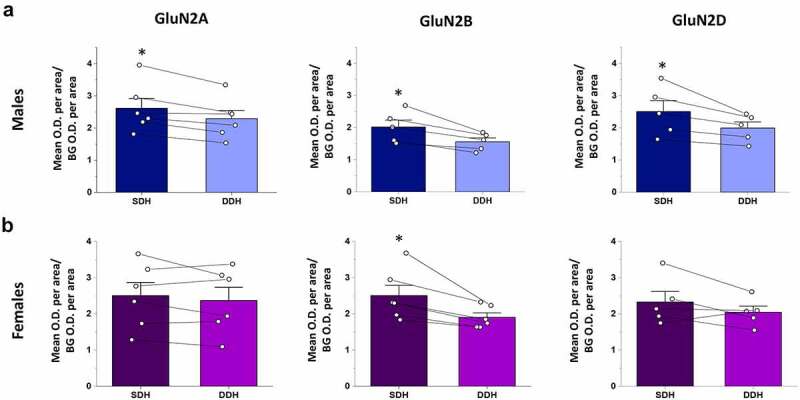
Relative expression of GluN2A, GluN2B and GluN2D in the SDH versus the DDH of males and female rats. Optical density of a given GluN2 signal per area for the SDH as well as for the DDH was normalized to background optical density per area (see box in Figure 2). Average SDH and DDH measures were derived from multiple spinal sections for each animal (shown as individual data points), followed by a paired comparison between the normalized SDH and DDH for each GluN2 subtype. (a) In males, there is significantly higher expression in the SDH (dark blue bars) versus the DDH (light blue bars) for all the three GluN2 subunits, but with more pronounced differential expression for GluN2B (middle) and GluN2D (right). (b) In females, the GluN2B isoform (middle) is the only GluN2 subunit that exhibits significantly greater expression in the SDH  (dark purple) versus the DDH  (light purple). *p < 0.05

We next quantified and compared the relative total staining intensity (per area) for each GluN2 isoform in the SDH and DDH. In male rats, expression of GluN2A, GluN2B and GluN2D subunits were significantly higher in the CGRP+ SDH versus the DDH (p = 0.012, n = 6 for GluN2A; p = 0.014, n = 5 for GluN2B; and p = 0.039, n = 5 for GluN2D; Figure 3(a)). The SDH/DDH relative expression ratio was greater for GluN2B (1.28 ± 0.05, n = 5) and GluN2D (1.24 ± 0.06, n = 5) isoforms compared to GluN2A (1.14 ± 0.03, n = 6), further supporting our observations of preferential localization of GluN2B and GluN2D to the SDH. However, ANOVA analysis revealed no significant difference (p = 0.11) between groups. It should be noted that GluN2B expression was also pronounced in the inner region of lamina II (Figure 2(a) middle), which is included as “DDH” as it is not part of the CGRP+ SDH (lamina I/II_O_) region, and so the quantified difference in SDH versus DDH expression for GluN2B would likely be even greater if lamina II_i_ was included in the quantified SDH region.

### Only the GluN2B isoform is preferentially localized to the SDH of female rats

In parallel with the above analysis in males, we studied the expression patterns of GluN2 subunits in the L4/L5 spinal cord of juvenile female rats. From a qualitative perspective, the relative intensity and localization of GluN2A, GluN2B, and GluN2D immunoreactivity across dorsal horn laminae were similar between males and females (Figure 2). In females, GluN2A immunoreactivity was diffusively distributed at neuropil, with robust and approximately equal staining intensity across the SDH and DDH (Figure 2(b), left and Suppl. Figure 2). GluN2B immunolabelling was also found throughout all laminae of the dorsal horn, but both cell bodies and neuropil exhibited GluN2B immunoreactivity (Figure 2(b), middle and Suppl. Figure 4). In terms of the overall intensity of GluN2B staining, there appeared to be a slightly stronger GluN2B signal in the CGRP+ SDH compared to the DDH. Unlike GluN2B, GluN2D immunoreactivity was similar in overall intensity across the SDH and DDH of female rats (Figure 2(b), right and Suppl. Fig. 6). This is a divergence from male staining, where GluN2D was preferentially localized to the SDH (Figure 2(a), right). However, the subcellular pattern of immunopositivity for GluN2D in female spinal cord was similar to that of GluN2B, with labeling of both neuropil and cell bodies (Figure 2(b) right, top).

As found through qualitative analysis, quantitative analysis revealed that only the GluN2B NMDAR isoform was preferentially localized to the SDH in female spinal cord. Statistical analysis showed no significant difference in staining intensity per area in the SDH versus the DDH for both the GluN2A (p = 0.35, n = 6) and GluN2D (p = 0.20, n = 5) isoforms, while there was significantly higher immunoreactivity for GluN2B in the SDH compared to the DDH (p = 0.020, n = 6; Figure 3(b)). Calculating the ratio of immunoreactivity in the SDH versus DDH revealed values near 1 for both GluN2A (1.07 ± 0.05, n = 6) and GluN2D (1.13 ± 0.09, n = 5), while GluN2B had a SDH/DDH immunoreactivity ratio of 1.30 ± 0.07 (n = 6). However, as for males, ANOVA analysis revealed no significant difference between groups (p = 0.085).

### Expression of GluN2 subunits across the mediolateral axis of the SDH: GluN2B immunoreactivity is enriched in the medial SDH of males

**Figure 4.**
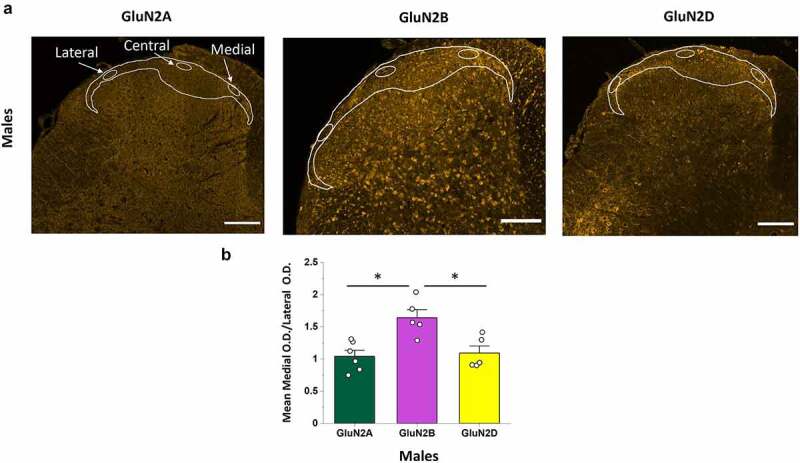
The GluN2B, but not GluN2A or GluN2D, isoform is preferentially expressed in the medial versus the lateral region of the SDH in male rats. (a) Representative confocal images (20x objective) illustrating the immunofluorescence for GluN2A (left), GluN2B (middle), and GluN2D (left). For each isoform, the OD per area was quantified in selected oval regions positioned within the medial, central and lateral regions of the CGRP+ SDH, as shown. There was a visible increase in intensity and distribution of fluorescent puncta in the medial SDH compared to the lateral SDH for GluN2B but not GluN2A or GluN2D. Scale bar: 200 µm. (b) Statistical analysis showing the mean medial vs lateral OD ratio for GluN2A, GluN2B and GluN2D. The medial/lateral OD ratio was significantly higher for GluN2B compared to both GluN2A and GluN2D. *p < 0.05

A recent voltage-sensitive dye imaging study in male rats has demonstrated that evoked NMDAR-dependent excitability is not equal across the mediolateral axis of the SDH [40]. To investigate the potential molecular underpinnings of this phenomenon, we first studied the relative expression of GluN2A, GluN2B, and GluN2D along the mediolateral axis of the CGRP+ SDH in male rats. The intensity of GluN2 immunoreactivity was measured in discrete regions of the medial, central, and lateral portions of the top-most CGRP+ SDH using a blinded approach where only the CGRP fluorescence signal was visible during placement of the small oval sampling windows (Figure 4(a), left). We found that the qualitative expression pattern for GluN2A (Figure 4(a), left) and GluN2D (Figure 4(a), right) immunoreactivity was visibly homogenous across the mediolateral axis. Surprisingly, GluN2B immunoreactivity appeared to be preferentially localized to the medial region of the CGRP+ SDH, with a fluorescence intensity that progressively declined into the central to lateral SDH regions (Figure 4(a), middle and Suppl. Figure 3). Quantitative analysis revealed a large, non-significant, reduction in the mean OD per area values from medial to central to lateral CGRP+ SDH for GluN2B (p = 0.099, n = 5), while OD values were highly conserved from medial to central to lateral SDH regions for both GluN2A (p = 0.23, n = 6), and GluN2D (p = 0.72, n = 5) (data not shown).

To further quantify potential differences in GluN2 expression across the mediolateral axis, we next compared the ratio between GluN2 immunoreactivity in the medial versus lateral sampled regions – the opposite ends of the dorsal horn where visible differences in intensity were greatest for GluN2B. We found that the medial/lateral OD ratio was near 1 for both GluN2A (1.04 ± 0.09, n = 6) and GluN2D (1.09 ± 0.11, n = 5), while GluN2B had a medial/lateral immunoreactivity ratio of 1.64 ± 0.12 (n = 5). This medial/lateral ratio for GluN2B was significantly higher than for both GluN2A and GluN2D (p = 0.003, n = 6, n = 5, n = 5; post hoc Tukey test GluN2B vs GluN2A: p = 0.004; GluN2B vs GluN2D: p = 0.011; Figure 4(b)).

### GluN2B mediolateral expression is not conserved between males and females

**Figure 5.**
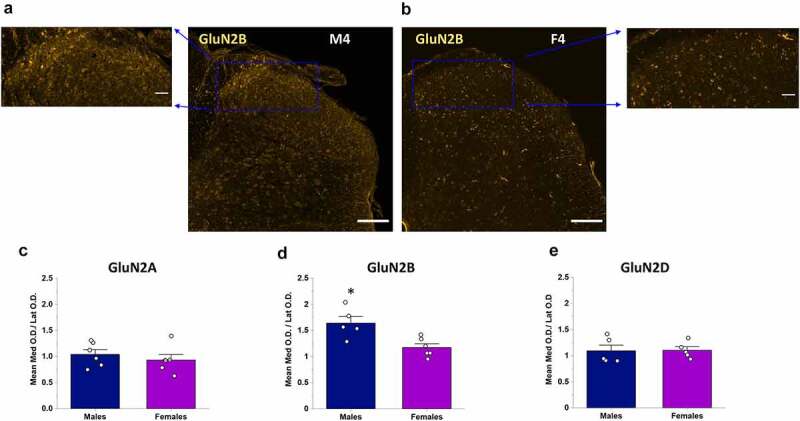
Comparison of the medial vs lateral OD ratio of GluN2 subunit isoforms between males and female rats. A, B) Representative confocal images showing GluN2B immunofluorescence in a male (a) and female rat (b), Scale bar: 200 μm, as well as higher magnification insets of the SDH for both male (top left) and female (top right), Scale bar: 50 μm. GluN2B signal is preferentially localized to the medial portion of the SDH in males (a) but not females (b). (c–e) Quantitative statistical analysis of the mean medial vs lateral OD ratio for GluN2A (c), GluN2B (d) and GluN2D (e) in males versus females. The medial/lateral ratio for GluN2B was significantly greater in males compared to females (d), while the medial/lateral ratio was not significantly different between males and females and near values of 1 for both GluN2A (c) and GluN2D (e). *p < 0.05

We next investigated whether the same mediolateral GluN2 expression patterns were observed in female spinal cord. At a qualitative level, there was no visible difference for GluN2A and GluN2D immunoreactivity across the mediolateral axis of the SDH, and only a moderately higher immunoreactivity for GluN2B in the medial SDH in a subset of female rats (Figures 2(b) and 5(b), Suppl. Figures 2, 4, 6). The measured OD per area were not statistically different between the medial, central, and lateral regions of the CGRP+ SDH for GluN2A (p = 0.45, n = 6), GluN2B (p = 0.15, n = 6), or GluN2D (p = 0.36, n = 5) subunits. Furthermore, in contrast to that observed in males, the medial/lateral OD ratio was not significantly different between GluN2A, GluN2B, and GluN2D subunits in females (p = 0.15; n = 6, n = 6, n = 5, respectively), with all ratios near a value of 1 (purple bars in Figure 5(c–e)).

To further test if the mediolateral expression patterns of GluN2 subunits are conserved between males and females, we directly compared the medial/lateral OD ratio for each subunit between sexes. This comparison reinforced the surprising finding that the specific localization of GluN2B to the medial SDH is present in male rats only (Figure 5). Qualitative visual evidence (Figure 5(a,b)) was confirmed by quantitative analysis showing a significant difference in the medial/lateral OD ratios for GluN2B between male and female rats (p = 0.008, Figure 5(d)). In contrast, the medial/lateral OD ratio was not significantly different between the sexes for both GluN2A (p = 0.46, Figure 5(c)) and GluN2D (p = 0.94, Figure 5(e)).

## Discussion

In this study, we used immunohistochemistry with antigen unmasking [17] to investigate the differential expression of specific GluN2-NMDAR subunits across the dorsal horn in male and female juvenile rats. We found that both GluN2B and GluN2D are predominantly localized to the SDH compared to the DDH in male rats, while only GluN2B is enriched in the SDH of females. In both sexes, GluN2A immunoreactivity is almost exclusively restricted to neuropil, while GluN2B and GluN2D are found in both the neuropil and the soma of dorsal horn neurons. Surprisingly, we discovered that GluN2 subunit expression is not equal across the mediolateral axis of the SDH, with a pronounced preferential expression of GluN2B in the medial SDH of male rats. In a further twist, this differential GluN2B expression was not observed in females, as all three GluN2 subunits were evenly expressed across the mediolateral SDH axis in female spinal cord. We, therefore, conclude that specific GluN2 subunits are differentially expressed across and within dorsal horn laminae, with clear sex-differences in these NMDAR subunit distribution profiles. These results have large implications for understanding potential differential roles of NMDARs in physiological and pathological pain processing between males and females.

Our finding that both GluN2B and GluN2D are preferentially expressed in the male SDH is consistent with previous reports demonstrating predominant localization of both subtypes to the SDH [17,22]. The high expression of both isoforms in the SDH is also consistent with functional evidence. Unlike the majority of mature CNS synapses where GluN2A dominates excitatory NMDAR-mediated responses [11], both GluN2B- and GluN2D-containing receptors mediate a significant fraction of synaptic responses in laminae I and II neurons of male rats [19,20,41]. As observed in other CNS regions [11], there is also evidence for the contributions of GluN2B- and GluN2D-containing receptors to extrasynaptic glutamate-evoked NMDAR responses in SDH neurons [18,23,24]. In agreement with single-channel recordings demonstrating GluN2B- and GluN2D-like currents on SDH somatic membranes [18], our results here show significant localization of both GluN2B and GluN2D protein to the soma of SDH neurons. Previous studies have reported a pronounced contribution for GluN2A-containing NMDARs to the afferent-evoked synaptic responses of SDH neurons [18,21,23,41], which is consistent with our finding of robust expression of GluN2A protein that is mainly confined to synaptic neuropil across the dorsal horn. Taken together, our experimental results on the relative cellular and subcellular localization of GluN2A, GluN2B, and GluN2D proteins across the dorsal horn of male rats align well with the available functional and immunohistochemical data from male and unspecified sex rodents.

Although the prevalence of chronic pain syndromes is higher in females [2], the vast majority of preclinical basic science research into molecular mechanisms of pain processing have been conducted in male rodents. This study highlights the urgent need to systematically characterize all elements of pain processing in female animal models, as even fundamental mediators of synaptic transmission and plasticity – NMDAR subunits – are differentially expressed at baseline between males and females. We found that the overall expression and subcellular localization pattern of GluN2A, GluN2B, and GluN2D in the dorsal horn were relatively conserved between males and females. However, unlike males, only GluN2B (and not GluN2D) was preferentially localized to the SDH in female spinal cord. Moreover, the preferential localization of GluN2B to the medial SDH was observed in males and not females (see below for importance). The underlying mechanisms that mediate this differential NMDAR subunit expression between sexes remain to be explored and could be due to hormonal, genetic, and/or epigenetic factors. For example, both estrogen receptor α (ERα) and estrogen receptor β (ERβ) are expressed in laminae I and II from an early embryonic stage and are involved in dorsal horn morphogenesis, such as the differentiation of specific neuronal subpopulations [42]. Interestingly, both ERα and ERβ are expressed within glial cells of the developing spinal cord [43], and thus, 17-β-estradiol (E2) may act on ER receptors within neurons or glia to directly or indirectly mediate transcriptional changes and differentiation within dorsal horn neurons. To further investigate this, it will be important to examine the potential differential expression of NMDAR subunits and their regulators within specific spinal subpopulations of neurons and glia in males versus females using single-cell sequencing approaches [16,44]. Given that this study was conducted in juvenile rats, future studies should also investigate NMDAR subunit expression in adult rats to determine whether puberty further enhances (or diminishes) the differential expression of NMDAR subunits between sexes.

One of the most striking effects of this study, which was initially identified through an unbiased quantitative analysis approach, is the pronounced localization of GluN2B to the medial SDH in male rats. This finding is consistent with cytoarchitectural and functional studies on juvenile rodents demonstrating dendritic asymmetry and enhanced NMDAR-dependent evoked excitability in the medial compared to lateral SDH [40,45]. It will be critical to test whether this same asymmetry along the SDH mediolateral axis persists into adulthood. If so, this heterogeneity would further support hypotheses based on single-cell sequencing [16,44] and neurochemical [46] results that differential neuronal circuits exist throughout the SDH and are devoted to specific aspects of sensory integration and processing. A critical experimental implication of our mediolateral GluN2B expression findings is that researchers should take careful note of where along the mediolateral axis they are studying and recording from when investigating the physiological and molecular properties of SDH neurons. From our results here and those of many other research groups, it is clear that the SDH does not consist of homogenous repeating neuronal circuits throughout its axes. Future research is needed to uncover the molecular underpinnings of these differences, especially given the large clinical implications of differences within these nociceptive circuits between males and females.

## Supplementary Material

Supplemental Material
