# Supplementary material for: Differential expression of GluN2 NMDA receptor subunits in the dorsal horn of male and female rats
Source: Channels (Austin). 2021 Jan 28;15(1):179–92. doi: 10.1080/19336950.2020.1871205 (PMC7849732; doi:10.1080/19336950.2020.1871205)
Supplement: Supplemental Material [file KCHL_A_1871205_SM1154.pdf]

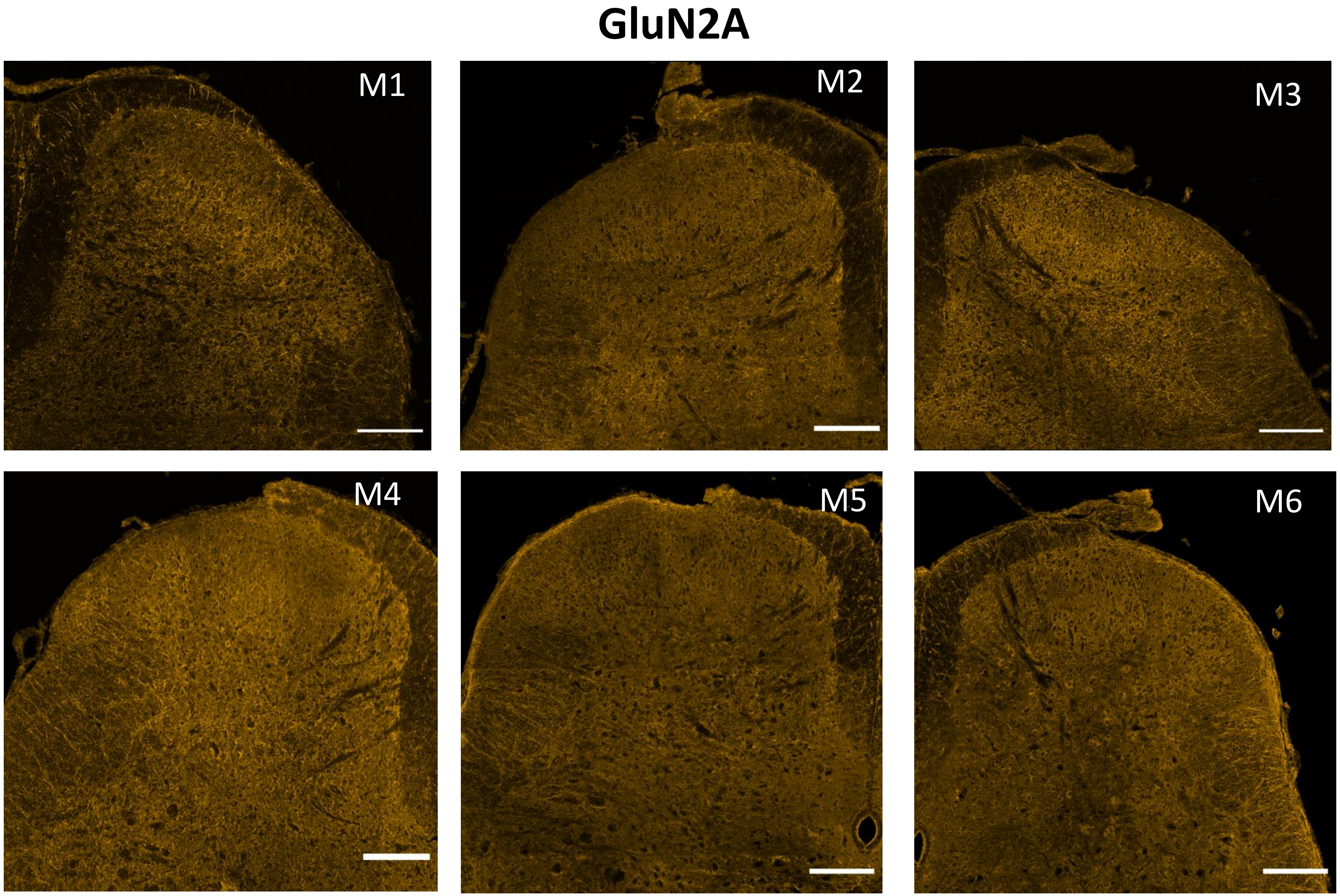

Supplementary Figure 1: Representative immunohistochemistry confocal images showing in yellow the immunoreactivity for GluN2A in male p21 rats. **Scale bar:** 200 µm

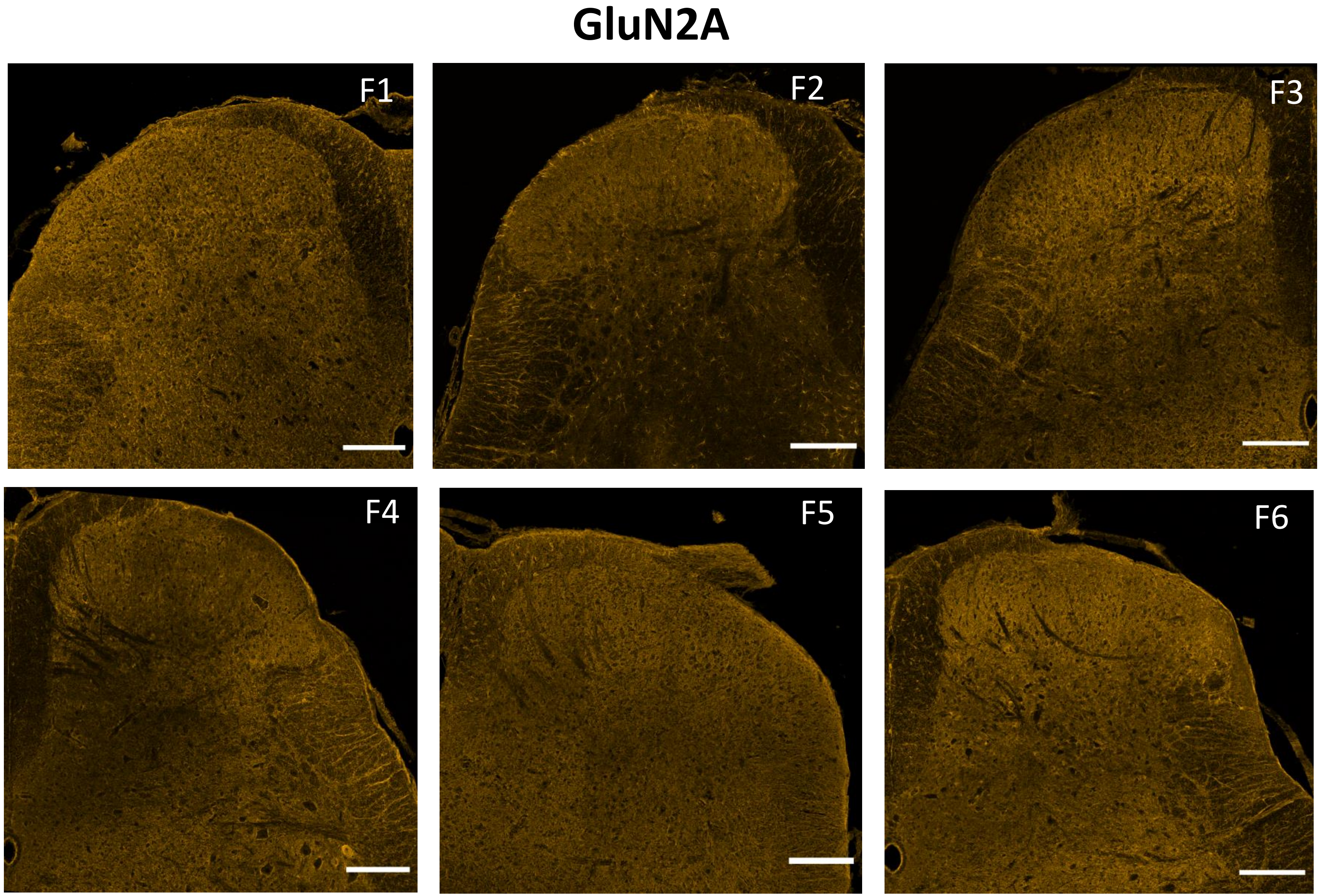

Supplementary Figure 2: Representative immunohistochemistry confocal images showing in yellow the immunoreactivity for GluN2A in female p21 rats. **Scale bar:** 200 µm

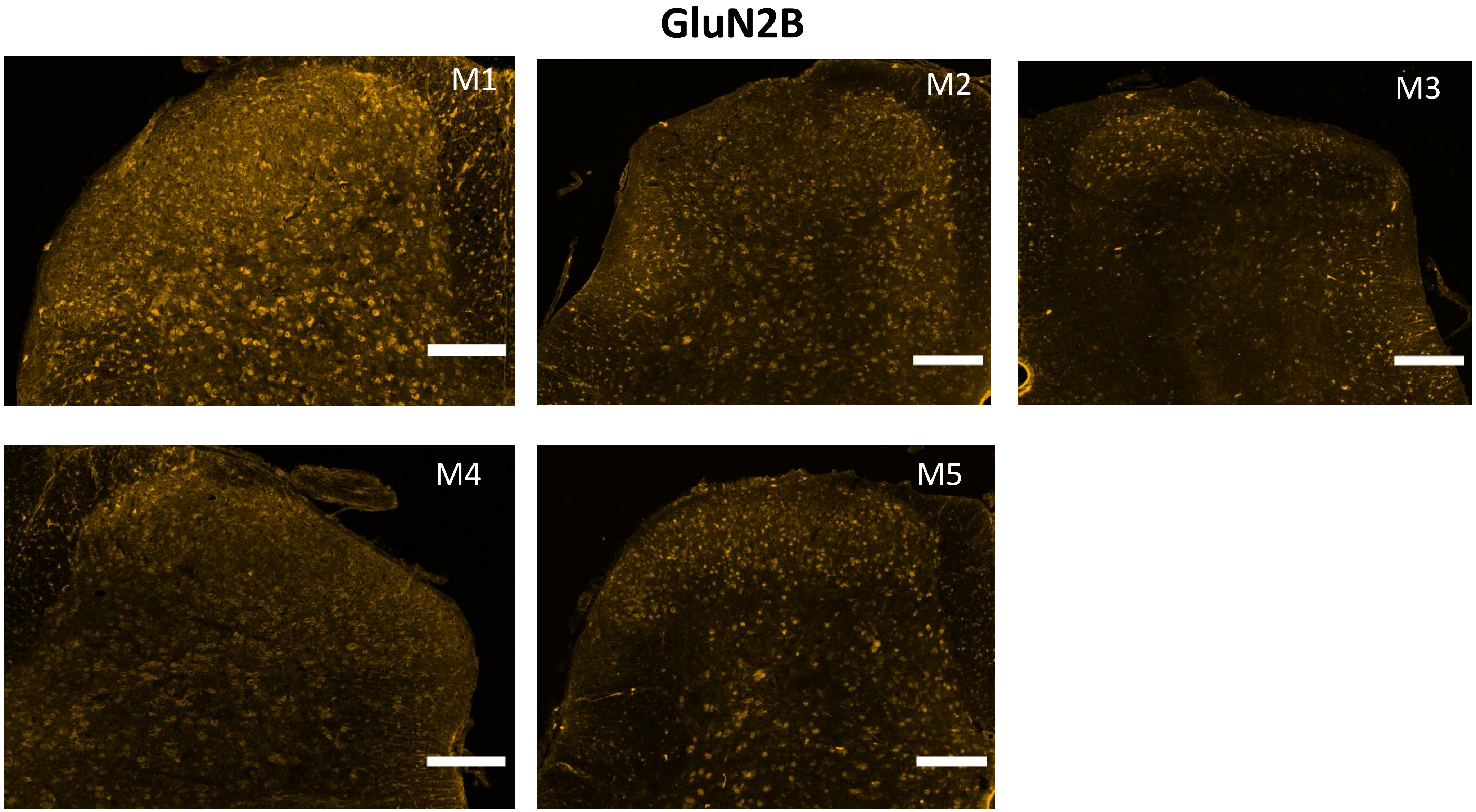

Supplementary Figure 3: Representative immunohistochemistry confocal images showing in yellow the immunoreactivity for GluN2B in male p21 rats. **Scale bar:** 200 µm

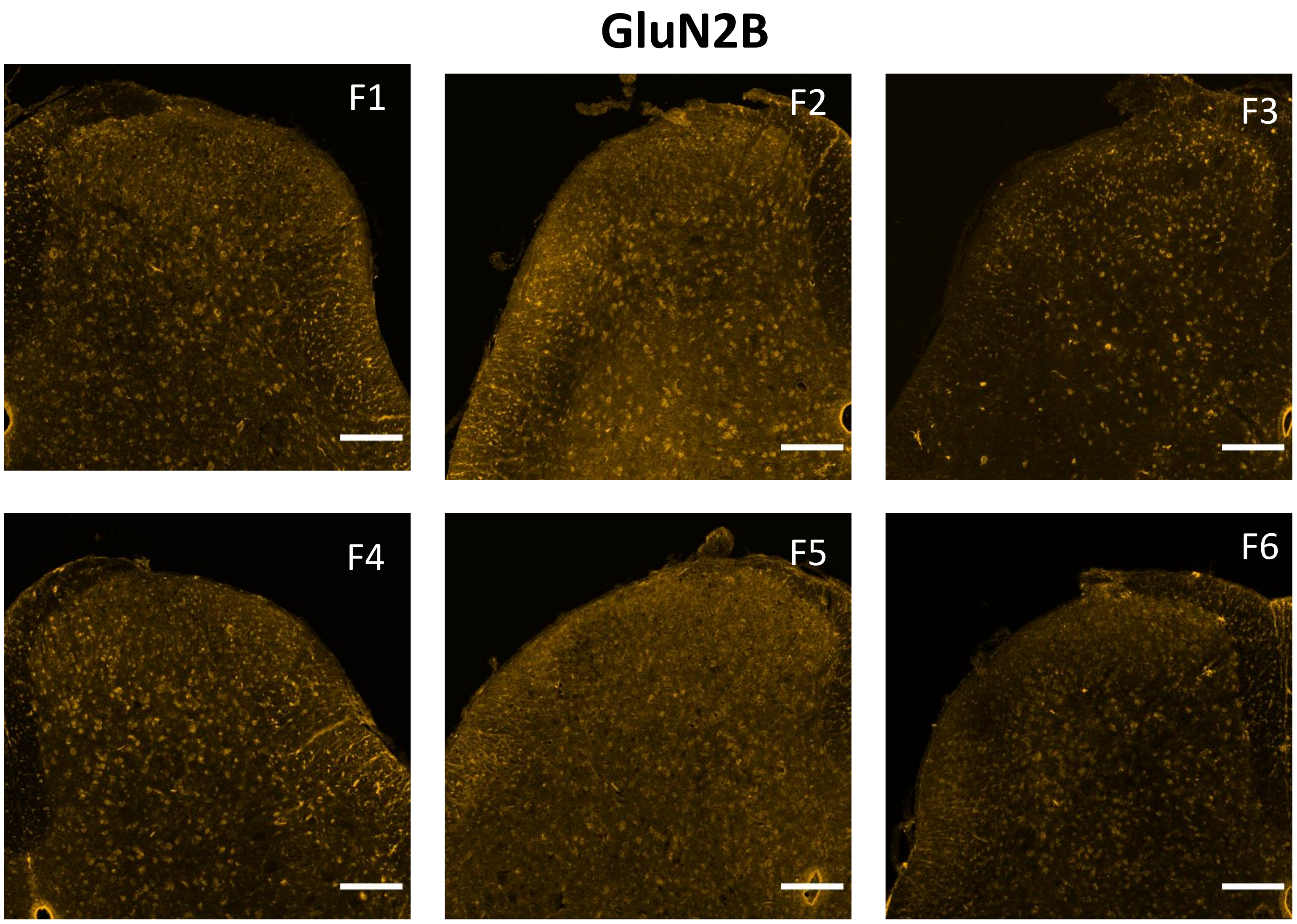

Supplementary Figure 4: Representative immunohistochemistry confocal images showing in yellow the immunoreactivity for GluN2B in female p21 rats. **Scale bar:** 200 µm

GluN2D

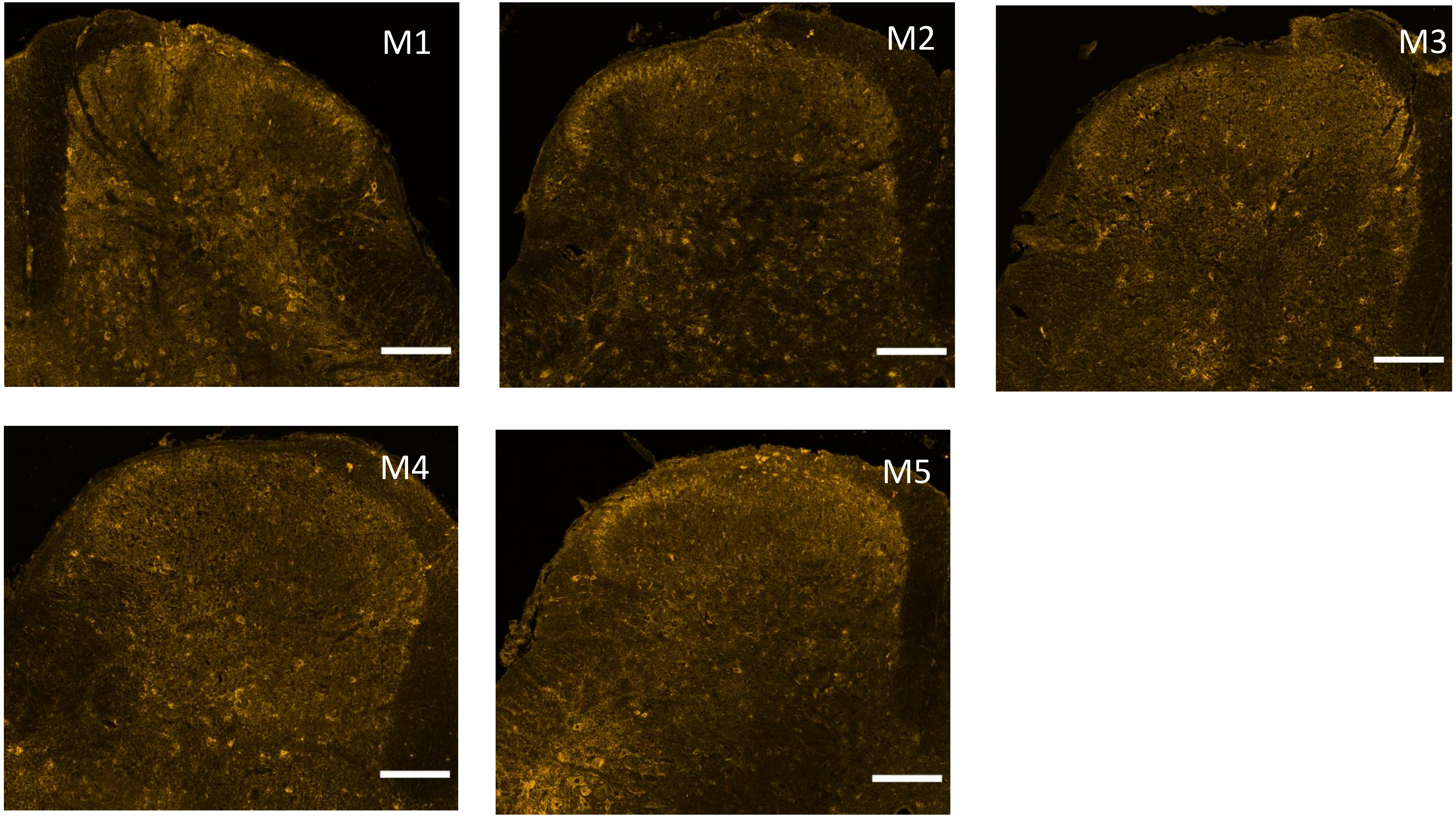

Supplementary Figure 5: Representative immunohistochemistry confocal images showing in yellow the immunoreactivity for GluN2D in male p21 rats. **Scale bar:** 200 µm

GluN2D

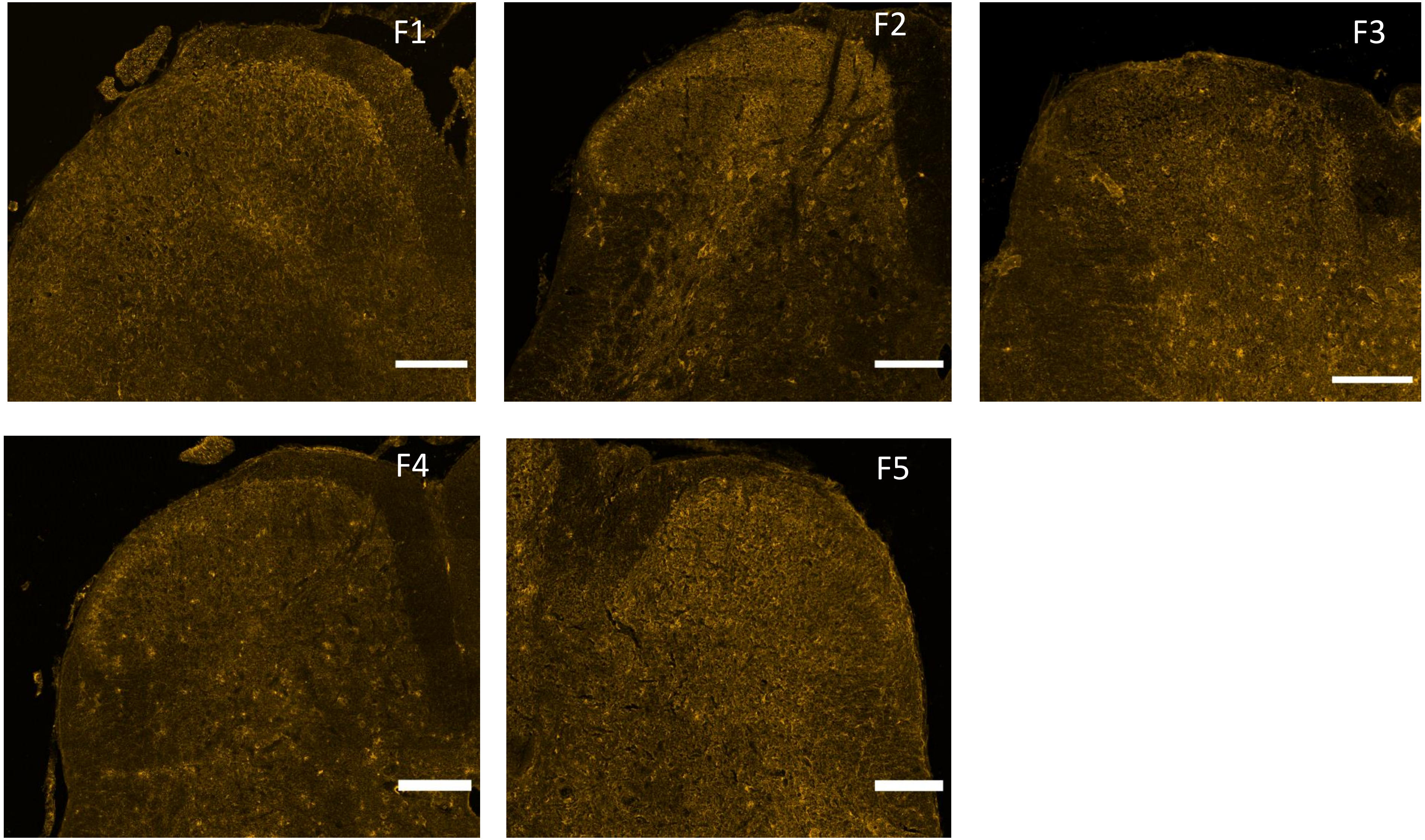

Supplementary Figure 6: Representative immunohistochemistry confocal images showing in yellow the immunoreactivity for GluN2D in female p21 rats. **Scale bar:** 200 µm
